# Gastric Rupture Secondary to a Heimlich Maneuver: A Case Report

**DOI:** 10.7759/cureus.66466

**Published:** 2024-08-08

**Authors:** Nathaniel Kleytman, Savni Satoskar, Cesar Riera Gonzalez, Abenezer Tedla, Nithyla John, Sania Thite, Daniel T Farkas

**Affiliations:** 1 General Surgery, BronxCare Health System, Bronx, New York, USA; 2 General Surgery, St. George's University School of Medicine, True Blue, GRD

**Keywords:** isolated gastric rupture, gastric burst injury, omental patch, heimlich maneuver, blunt abdominal injury, blunt abdominal trauma, stomach perforation, lesser curvature, gastric perforation, gastric rupture

## Abstract

A gastric rupture resulting from abdominal trauma is a rare occurrence with a frequency of less than 2% of blunt abdominal injuries. Furthermore, gastric perforation secondary to the Heimlich maneuver is even rarer, with only a handful of cases reported in the literature. Here, we present a case of isolated gastric rupture following a successful Heimlich maneuver. Laparotomy revealed a large perforation along the lesser curvature of the stomach. The perforation was repaired by primary closure and reinforced with omental patching.

## Introduction

The commonly recommended Heimlich maneuver is a popular technique within the first-aid repertoire to relieve foreign object obstruction of the airway. In 1974, Henry Heimlich described the method whereby a rescuer exerts a rapid and forceful upward thrust to a choking victim’s abdomen compressing the diaphragm and lungs, generating significant positive intrathoracic pressure aimed at expelling a food bolus [1]. Although universal in use, the Heimlich maneuver is not free of consequences and can ironically lead to several rare but life-threatening injuries. Here, we report a case of gastric rupture following a successful Heimlich maneuver. We outline the steps taken to ensure the proper diagnosis and management of this rare cause of stomach perforation and provide a review of the relevant literature on perforations secondary to blunt or iatrogenic abdominal trauma.

## Case presentation

A 55-year-old male was brought to the emergency department for evaluation of increasing epigastric pain of several hours duration. History revealed that earlier the same day, the patient was eating a pastrami sandwich at which point he began choking. The patient’s wife administered back blows to no avail, consequently proceeding to administer the Heimlich maneuver. After this, she was able to use a finger sweep to clear the obstructing food bolus, at which time he began breathing again. Soon after this, the patient began to experience epigastric pain with increasing abdominal distension. Past medical history was significant for hypertension and remote traumatic brain injury as a result of a gunshot wound, with subsequent seizure disorder and schizoaffective disorder.

In the emergency room, the patient was hemodynamically stable with normal vital signs. His blood pressure was 121/73 with a heart rate of 85. He had only mild discomfort and was not in significant distress with an oxygen saturation (SpO_2_) of 95% on 2L/min of oxygen administered via nasal cannula. Physical exam was remarkable for diminished breath sounds in the right lower lung field and diffuse tenderness and distention of the abdomen. There was minimal guarding, with no rigidity.

**Figure 1:**
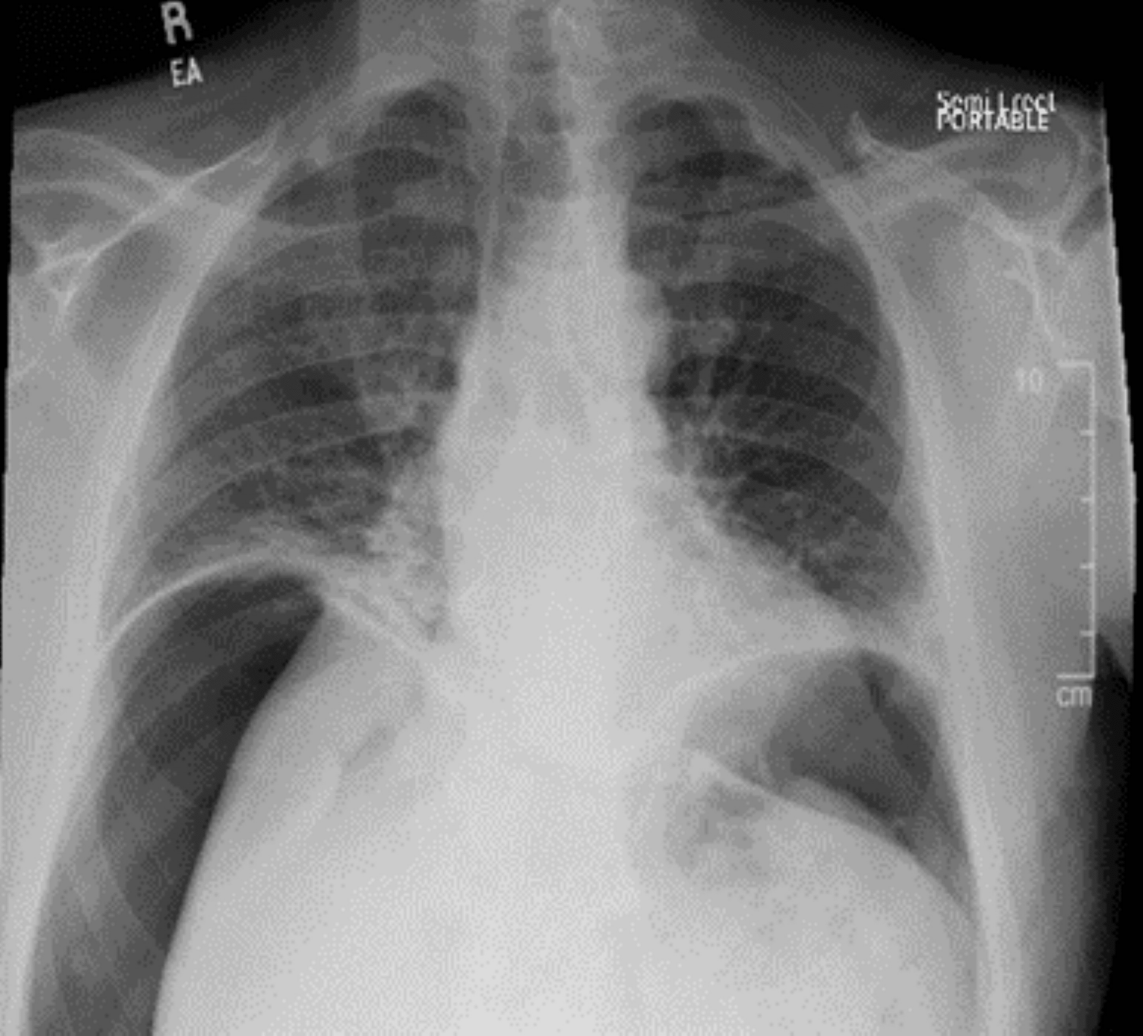
Chest x-ray showing pneumoperitoneum with bilateral subdiaphragmatic air

The patient’s white blood cell count was 10,300/µL with 85.7% being neutrophils. Mild elevation of blood urea nitrogen levels to 28 mg/dL with creatinine 1.5 mg/dL. Lactic acid was 1.2 mmol/L, within the normal range. A chest X-ray showed significant bilateral subdiaphragmatic air (Figure 1).

**Figure 2:**
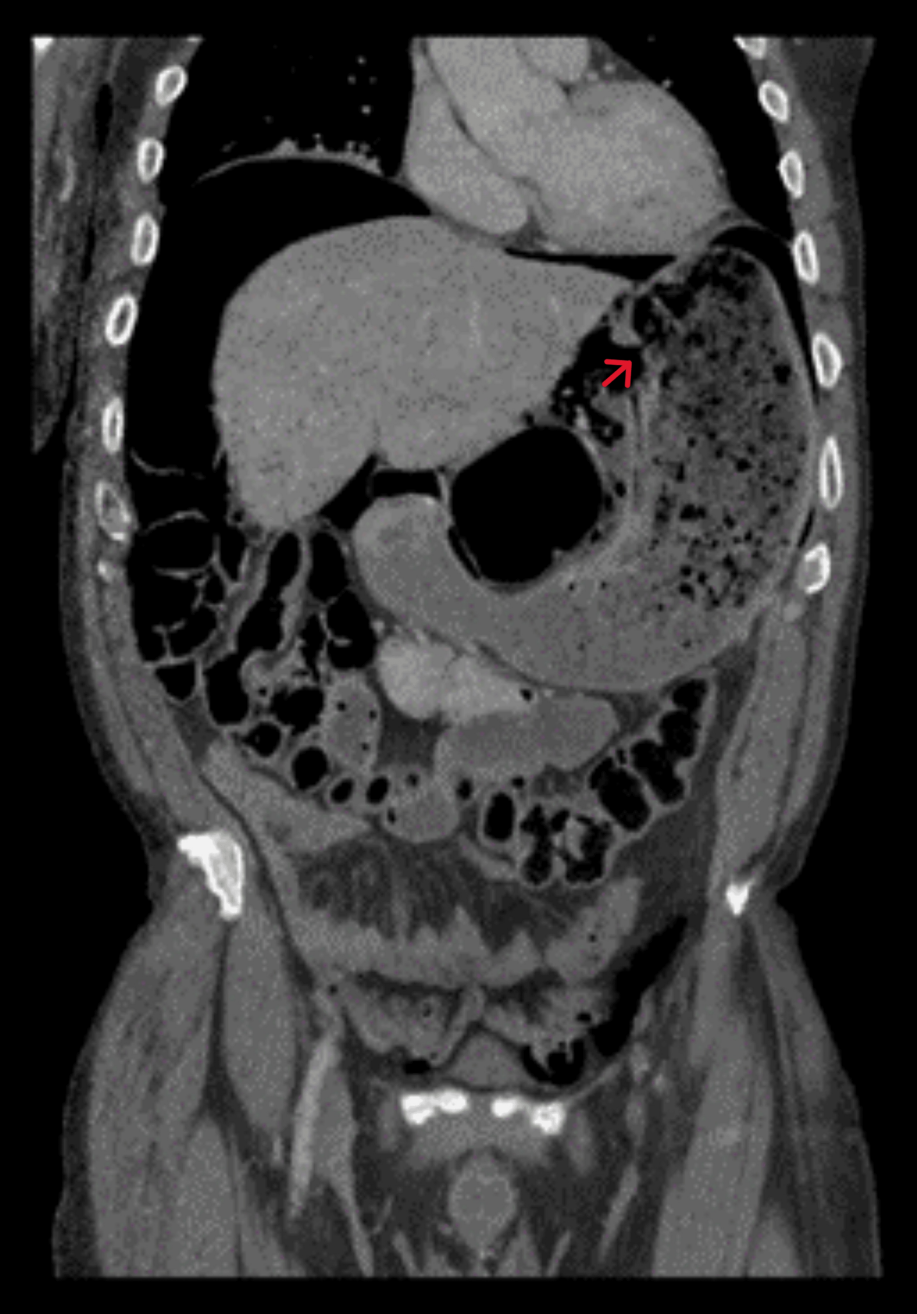
Coronal view on CT showing extensive pneumoperitoneum and discontinuity of the stomach wall (red arrow), indicative of gastric rupture

A CT scan was done and showed a large amount of pneumoperitoneum. The stomach was noted to be distended and full of food, with discontinuity of the gastric wall seen on the lesser curvature (Figure 2). Mild bibasilar atelectasis was observed as well.

The patient was transferred to the surgical critical care unit. He received two liters of Ringer’s lactate solution, intravenous piperacillin-tazobactam, fluconazole, and pantoprazole. A nasogastric tube was placed for gastric decompression and the patient was scheduled for emergency exploratory laparotomy.

**Figure 3:**
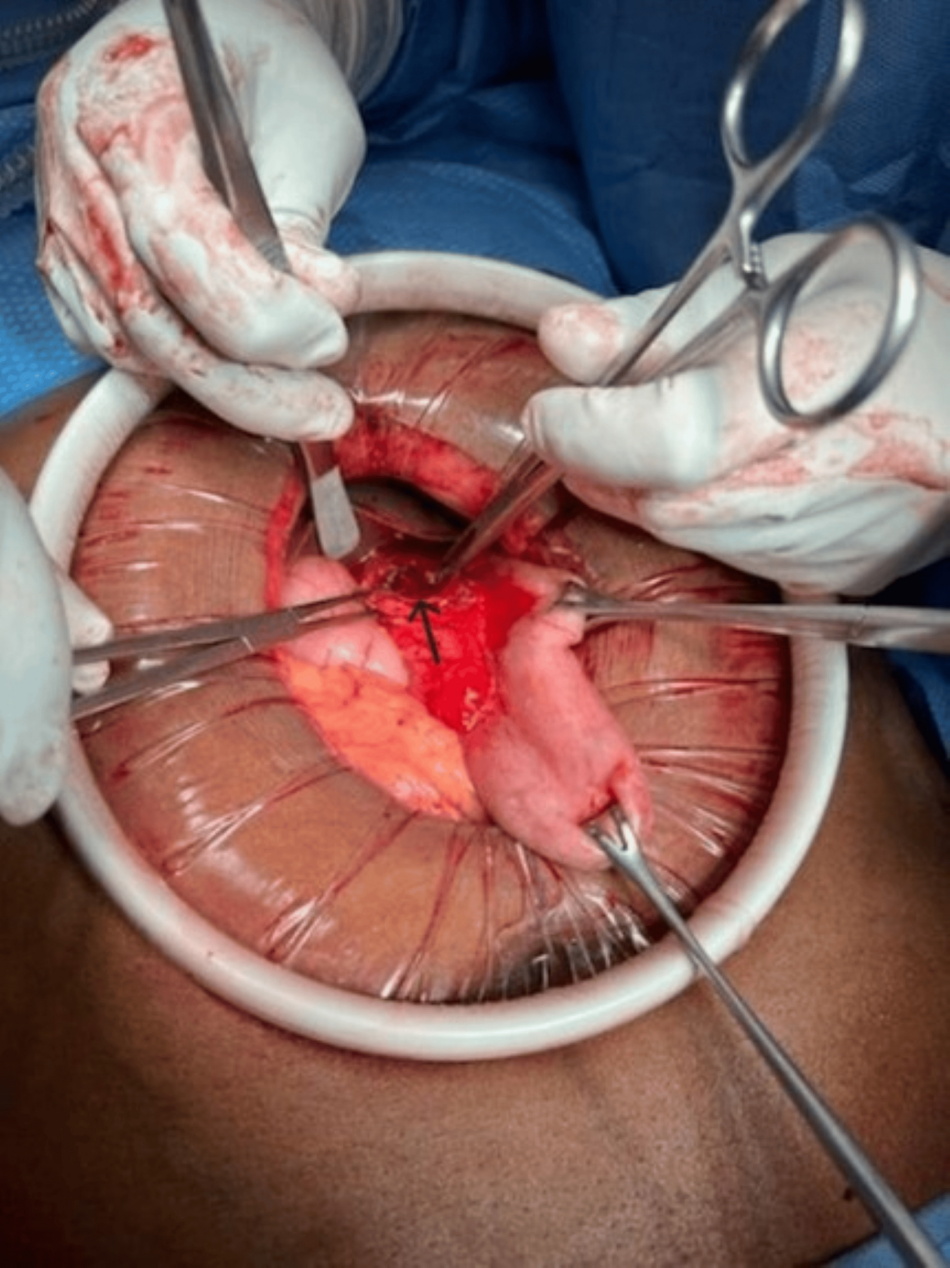
Intraoperative picture showing perforation (black arrow) along the lesser curvature

Upon midline incision, there was an immediate release of a large amount of pressurized air from the peritoneum. There was no free fluid seen. Ecchymosis was seen along the anterior surface of the lesser curvature extending into the lesser omentum. Air was insufflated into the stomach and a leak was identified. The lesser omentum was divided and trimmed back to expose the stomach. There was a large, full-thickness burst injury along the lesser curvature of the stomach with an avulsion of the overlying serosa off of the mucosa, leaving the tissue planes significantly disrupted (Figure 3).

**Figure 4:**
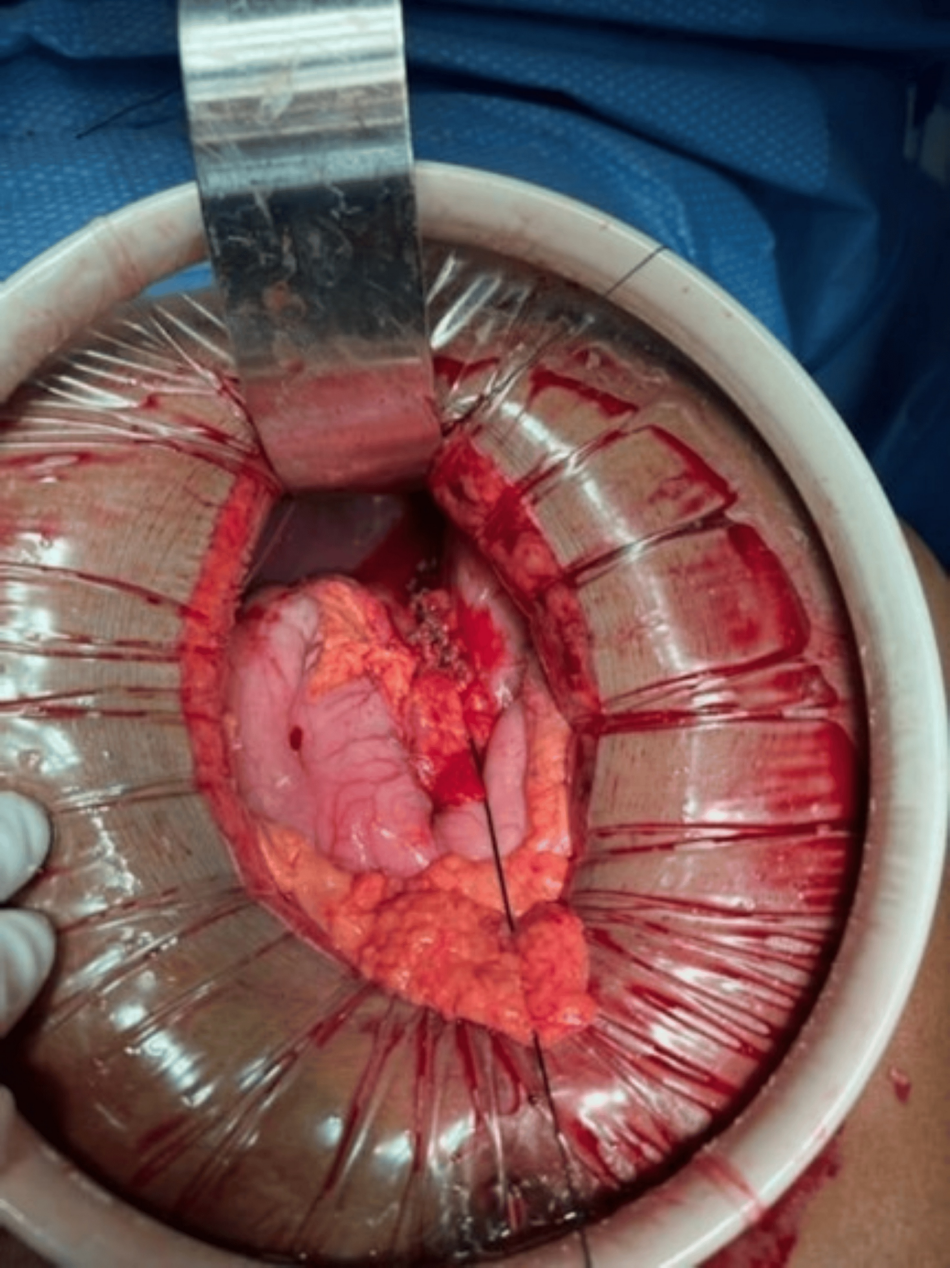
Intraoperative picture showing full-thickness, single-layer, longitudinal closure of the perforation

There was also a small degree of localized contamination with gastric contents, which was carefully suctioned out. Using suction, the remaining stomach contents were also removed. The edges of the tissue were all viable, and these were closed using a single layer of full thickness 2-0 silk sutures, closing the defect longitudinally (Figure 4).

The stomach was re-insufflated, and at this time, there was no further leak. After irrigation, a tongue of omentum was created using a LigaSure device (Medtronic plc, Dublin, Ireland), and this omentum was layered over the repair and secured with additional 2-0 silk sutures. The abdomen was thoroughly irrigated and gently suctioned out. A 15 FR Blake drain was placed and the abdomen was closed.

There were no postoperative complications and recovery was unremarkable. The nasogastric tube was left to low suction, and the drain remained serosanguinous. On postoperative day 4, an upper GI series was performed with no evidence of leakage from the stomach. The nasogastric tube was removed, and the patient was started on a clear liquid diet. This was advanced to a full liquid diet the next day, and the patient was discharged on postoperative day six. He was seen in the office for a follow-up after two weeks and was doing well, reporting one episode of mild epigastric pain. The patient’s abdomen was soft and non-tender with a well-healed incisional scar. One month of delayed-release pantoprazole was prescribed. He was seen again two months after surgery and was doing well without any complaints.

The patient and his wife agreed for this case to be published in the literature, and provided written consent.

## Discussion

First described by Henry Heimlich in 1974 after a series of animal experiments, the widely popularized Heimlich maneuver has become a mainstay first-aid technique for immediate relief of airway obstruction in a choking subject. The Heimlich maneuver involves exerting a swift, forceful upward thrust to the diaphragm and upper abdomen, generating positive intrathoracic pressure aimed at expelling an obstructive foreign object from the airway [1]. However, if the pressure is excessive or improperly directed, this blunt abdominal trauma can lead to a rapid increase in intra-abdominal pressure resulting in potentially life-threatening consequences [2]. Considering the stomach is a relatively hollow organ, positioned centrally immediately beneath the diaphragm with its distal half left unprotected and exposed anteriorly, the organ can be especially vulnerable to abrupt changes in pressure. This phenomenon is further magnified if the stomach is distended with food [3] and by the organ’s relative immobility within the peritoneum when taking into account its attachments by the lesser omentum, greater omentum, gastrophrenic ligament, gastrolienal ligament, and phrenicocolic ligaments. These biomechanical factors and rapid compressive forces from blunt abdominal trauma translate into exceedingly great intra-luminal pressures and shearing forces, which in tandem override the tension limits of the organ wall culminating in a brute burst force rupturing the stomach.

Gastric rupture following abdominal trauma is a rare occurrence with a reported frequency of 0.04-1.2% [4], far behind the prevalence of splenic and liver injuries. When considering abdominal traumatism, a case series examining thousands of patients at a single surgical department over several decades found only 25 cases of gastric laceration. The most common site of injury was the anterior wall followed by the greater curvature [5]. A review of a Japanese cohort revealed similar results in the anatomical distribution of injury [6]. Ueda et al. showed a predominance of isolated gastric rupture in 26 patients with blunt abdominal trauma. The authors posited the stomach serves as an airbag absorbing the force of impact and protecting the liver and spleen among other abdominal contents. In contrast, a review of 14 patients demonstrated most abdominal trauma patients had multiorgan injuries in addition to gastric perforation [7]. Similarly, an analysis of 33 gastric injury cases across multiple surgical centers in Brazil revealed only two cases of isolated gastric injury [8].

Gastric perforation as a complication of the Heimlich maneuver is even rarer with only a handful of cases reported in the literature [2,3,9-11]. Naturally, it is thought that the most common complications of the Heimlich maneuver are rib fractures and pneumomediastinum; however, a systemic review of 51 case reports found gastric injury most frequently occurred in 20% of patients who received the Heimlich maneuver [12]. The authors specified that of these cases, most occurred in the elderly and involved injuries particularly to the lesser curvature of the stomach. This is in agreement with the literature we reviewed as well. Darke and Bloomfield explained this phenomenon due to the lesser curve having relatively few mucosal folds and limited elasticity [13]. In concordance, here, we report a full-thickness burst injury to the lesser curvature of the stomach with ecchymosis extending into the lesser omentum.

After an extensive literature review, primary one-layer and two-layer closures are overwhelmingly the method of choice for surgical repair of traumatic gastric perforation. In a Spanish cohort of 25 patients, 22 had undergone simple suturing of the gastric lesion with the rest needing partial gastrectomy [5]. In a Japanese cohort of 26 patients, simple closure was used in nineteen patients with the rest receiving gastrectomy or conservative management [6]. Laparoscopy is not well studied in this exact scenario, as it is quite rare. However, the laparoscopic approach has been successful in treating perforations due to ulcer [14], cancer [15], and foreign body [16]. In addition, there is a case report of laparoscopy being used to treat a traumatic perforation secondary to cardiopulmonary resuscitation [17]. The use of an omental patch for perforation repair was reported in a handful of case reports with no postoperative complications [9,18]. During the exploratory laparotomy of our patient with gastric rupture, we elected to use a primary closure with the addition of an omental patch to increase effective wound healing. This is due to the omentum’s inherent vascular and nutritional supply creating a microenvironment to aid in the healing of epithelial and connective tissue cells [19].

## Conclusions

We report a rare case of gastric rupture of the stomach following a successful Heimlich maneuver. During laparotomy, a large perforation was identified along the lesser curvature. This is typical in cases of gastric rupture from blunt trauma. These can be repaired in one or two layers, with or without the addition of an omental flap. This case underscores the importance of medical evaluation following the Heimlich maneuver as well as physician awareness of such injuries despite their rare nature. Prompt surgical treatment is necessitated to avoid the development of peritonitis and spare the patient from septic shock.
